# 5‐Hydroxymethyl Furfural Oxidation by Perylene Diimide‐Sensitized Electrodes Boosted by Photoinduced Doping

**DOI:** 10.1002/cssc.202401782

**Published:** 2024-11-12

**Authors:** Edoardo Marchini, Stefano Carli, Davide Barboni, Martina Catani, Alberto Cavazzini, Stefano Caramori, Serena Berardi

**Affiliations:** ^1^ Department of Chemical Pharmaceutical and Agricultural Sciences University of Ferrara 44121 Ferrara Italy; ^2^ Department of Environmental and Prevention Sciences University of Ferrara 44121 Ferrara Italy; ^3^ Council for Agricultural Research and Economics-CREA 00184 Rome Italy; ^4^ National Interuniversity Consortium of Materials Science and Technology (INSTM) University of Ferrara Research Unit 44121 Ferrara Italy

**Keywords:** Antimony-doped tin oxide, Perylene diimide, HMF oxidation to FDCA, Photoinduced doping, Dye-sensitized photoanode

## Abstract

We explored the electrochemical behavior of antimony‐doped tin oxide (ATO) and perylene diimide (**PDI**)‐sensitized ATO (ATO‐**PDI**) for the (2,2,6,6‐tetramethylpiperidin‐1‐yl)oxyl (TEMPO) mediated conversion of 5‐hydroxymethyl furfural (HMF) to 2,5‐furandicarboxylic acid (FDCA), a value‐added substrate for alternative polymer synthesis. We first showed that ATO displayed good electrocatalytic properties towards TEMPO, affording a quasi‐reversible response with a heterogeneous rate constant on the order of 2×10^−4^ cm s^−1^. We then evaluated the performance of ATO under exhaustive electrolysis of HMF in basic aqueous electrolyte, reaching 80 % Faradaic Efficiency (FE) for FDCA production. Interestingly, a significantly enhanced current (up to 2.5 mA cm^−2^) was recorded over time when ATO‐**PDI** was exposed to prolonged visible illumination in a Dye‐Sensitized Photoelectrochemical Cell (DSPEC) configuration, which we ascribed to the photoinduced doping of ATO resulting from the oxidative quenching of **PDI** excited states. The proposed system enabled the production of FDCA with ca. 75 % FE in <2 h reaction time, and an almost quantitative HMF conversion when both the mono‐ and di‐acid products were considered. To the best of our knowledge, this is the first example of a molecular dye‐sensitized interface used for the TEMPO‐mediated oxidation of HMF.

## Graphical abstract

A molecular‐based photoanode, assembled from Sb‐doped tin oxide and a perylene diimide dye, promoted the oxidation of 5‐hydroxymethyl furfural (HMF) mediated by TEMPO radical. This system enabled the production of 2,5‐furandicarboxylic acid (FDCA), a possible replacement for terephthalic acid, with 80 % faradaic efficiency and current densities up to 2.5 mA cm^−2^ in <2 h reaction time.

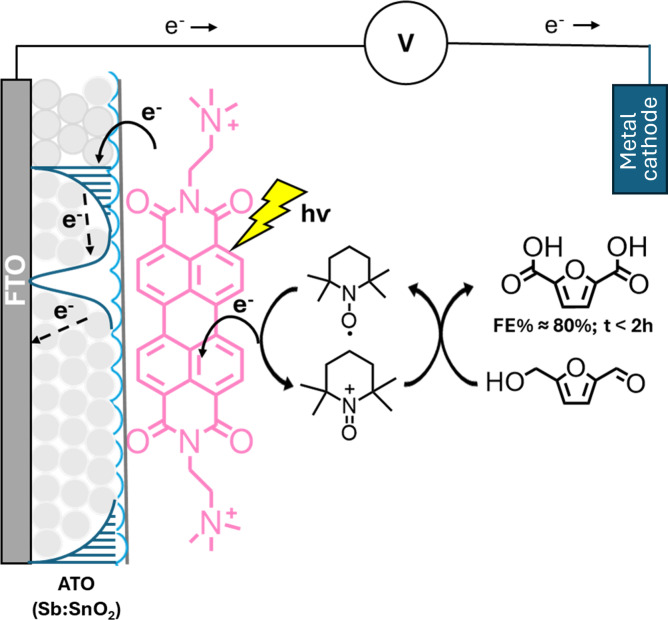

## 1 Introduction

Nowadays, the scientific community is actively working to mitigate the global warming caused by the anthropogenic activity. Among various approaches, researchers have been investigating light‐driven processes with the aim of storing solar energy into chemical bonds. In this framework, Dye Sensitized Photoelectrochemical Cells (DSPECs)^[1]^ stand out as a promising class of devices that mimic natural photosynthesis in promoting a cascade of charge transfer processes following the light‐induced generation of an electrochemical potential gradient. In the specific, in DSPECs sunlight absorption triggers the population of a dye′s excited state, able to undergo charge injection into a semiconductor′s conduction band. The reduced chromophore is successively regenerated through the oxidation of target substrates. Since the earliest contribution by Mallouk and coworkers,^[2]^ DSPECs have been mainly explored for water splitting, but alternative energy storage pathways, based on different oxidative chemistry routes, have been recently reported, including hydrobromic acid splitting,[^3^, ^4^, ^5^] and organic oxidations targeting carbonyl compounds^[6]^ or biomass valorization.[^7^, ^8^, ^9^, ^10^] Within this framework, the oxidation of 5‐hydroxymethylfurfural (HMF) to 2,5‐furandicarboxylic acid (FDCA) mediated by the (2,2,6,6‐tetramethylpiperidin‐1‐yl)oxyl radical (TEMPO) stands out, since FDCA can be used as a possible replacement for terephthalic acid for the synthesis of polyethylene terephthalate.[^11^, ^12^, ^13^] However, the alkaline conditions required to perform HMF oxidation pose challenges to DSPECs utilization, due to the hydrolysis of the ester bond usually linking the metal oxide semiconductors and sensitizers bearing traditional anchoring moieties (−COOH, −H_2_PO_4_).[^14^, ^15^, ^16^, ^17^, ^18^] To address this issue, some of us have successfully assessed the chemical stability of hydrophobic aggregates of a perylene diimide‐based dye, namely [(N,N′‐bis(2‐(trimethylammonium)ethylene)‐perylene‐3,4,9,10‐tetracarboxylic acid bis‐imide)](PF_6_)_2_ (**PDI**), over a wide range of pH (from 1 to 13) when supported on Antimony‐doped Tin Oxide (ATO) electrodes.^[19]^ In the resulting ATO‐**PDI** interfaces, an improved electron collection under sufficiently anodic bias was observed, resulting in 4 mA cm^−2^ photocurrent density for the photoinduced HBr splitting,^[20]^ due to the presence of intraband gap (IG) states, able to store photogenerated charges in long lived states. The promising stability and performances exhibited by ATO‐**PDI** photoanodes prompted us to investigate such interfaces also for the TEMPO‐mediated HMF oxidation in basic aqueous media. Indeed, examples of HMF oxidation performed in (photo)electrochemical devices only involve the use of medium/high band gap semiconductors,[^21^, ^22^, ^23^] or dark anodes.[^24^, ^25^] In particular, a 20–25 % current enhancement was recorded when ATO‐**PDI** was exposed to prolonged visible illumination, with respect to otherwise identical dark conditions. This likely originates from a photoinduced doping of the semiconductor resulting from the oxidative quenching of **PDI** excited states, which translates into an increased conductivity of the ATO film. Overall, we have successfully achieved a highly selective electrochemical oxidation of HMF to FDCA with 80 % Faradaic Efficiency (FE%) in a DSPEC configuration using ATO‐**PDI** photoanodes, enabling for a 20 % shorter reaction time with respect to the non‐sensitized system. To the best of our knowledge, this is the first proof‐of‐concept example of HMF oxidation photochemically enhanced by a sensitized interface working in basic aqueous electrolytes. Thus, our work contributes to widen the scope of application of DSPEC systems, albeit further optimization is needed to meet current densities relevant to large‐scale FDCA production.

## Materials and Methods

### Materials

ATO powder (Alfa Aesar NanoArc®, 99.5 %, Sb_2_O_5_:SnO_2_ 15 : 85 wt %, 13–22 nm powder), SnO_2_ colloidal solution (15 % in water), polyethylene glycol (PEG) 600 and 0.180 mm thick Nafion‐117 membrane were purchased from Alfa Aesar. Boric Acid, Acetonitrile (ACN) and Methanol HPLC grade were bought from Carlo Erba. 99.9 % titanium(IV) chloride_,_ Acetic Acid, Sodium Formate and Formic Acid HPLC grade, Alconox, ACS‐grade and 2‐propanol ≥99.8 % were obtained from Merck. TEC 7 Fluorine‐doped Tin Oxide (FTO) conductive glass slides were purchased from NSG. 5‐hydroxymethyl furfural (HMF) and 2,2,6,6‐Tetramethylpiperidine 1‐oxyl free radical (TEMPO) were acquired from Fluorochem. SnO_2_ and ATO colloidal paste were prepared according to literature procedure.[^20^, ^26^] Ultra‐pure water was obtained through a Milli‐Q‐ system (Millipore, USA).

### Photoanode Preparation

FTO slides were cleaned by subsequential immersion and sonication in Alconox solution and in 2‐propanol for 10 min each time, followed by 20 min annealing at 450 °C in a furnace under air atmosphere. An ultrathin compact underlayer of TiO_2_ was obtained on FTO by drop casting and slow hydrolysis of an aqueous 0.4 M TiCl_4_ solution in a close chamber for 12 h at RT. The resulting blocking underlayer was annealed at 450 °C for 30 min affording TiO_x_/FTO electrodes.

ATO′s porous semiconductor scaffold, was obtained by 5 successive spin coating cycles (first 6 s spinning at 600 RPM, then 20 s spinning at 2000 RPM) of the colloidal paste onto TiO_x_/FTO, with each casting followed by 550 °C annealing in air for 30 min in a pre‐heated muffle oven.

SnO_2_ electrodes were prepared by doctor blading the SnO_2_ colloidal precursor on top of TiO_x_/FTO electrodes, followed by 550 °C annealing for 30 min.

Electrode sensitization was realized by soaking the semiconductor films in 1 mM **PDI**/acetonitrile solution overnight. The resulting dyed electrodes were washed with fresh acetonitrile to remove excess of dye and then dried under air flow.

### Spectroscopic and Electrochemical Characterizations

Profilometry Analysis of the ATO‐ and SnO_2_‐based electrodes was performed with an Alpha step D‐500 Profilometer (KLA instruments, Milipitas, CA, USA). Data were collected in step‐up/down mode at a speed of 0.07 mm s^−1^ with a stylus force of 5.0 mg.

Absorption spectra of dyed electrodes were recorded against an undyed photoanode by employing an Agilent Cary 300 spectrophotometer. The same instrument was employed to collect the spectra of the solution before and after the electrolysis. Spectra were collected against water as reference.

Fourier transform infrared‐attenuated total reflection spectroscopy (FTIR‐ATR) of the **PDI** powder was performed with a Nicolet iS50 spectrometer with a 45° single‐reflection diamond ATR element. FTIR spectra of the bare ATO‐ and ATO‐**PDI**‐based electrodes were collected in external reflectance mode by means of a Seagull variable angle reflection accessory (Harrick Scientific, Inc. ).

Electrochemical characterization by means of cyclic voltammetry (CV), in the presence of TEMPO and HMF, was carried out with a PGSTAT 302 N potentiostat in a one‐compartment three‐electrode cell, by using ATO films or glassy carbon (GC) as working electrode, saturated calomel electrode (SCE) as reference and Pt wire as counter electrode. Unless otherwise stated, all potential values are given versus the saturated calomel electrode (SCE) throughout the paper. For the sake of comparison with other literature results in the field, the applied potentials versus the reversible hydrogen electrode (RHE) are also reported as the top‐*x* axis or in the legends. These values are calculated as follows:

(1) V(V)vsRHE=V(V)vsSCE+0.24+(0.059·pH)

The electrochemical reaction mechanisms were simulated with KISSA‐1D, according to the mechanisms reported below.[^27^, ^28^, ^29^, ^30^, ^31^] The electrochemical activity of ATO′s IG states was investigated by means of cyclic voltammetry in 0.5 M borate buffer pH 9 by scanning the potential in the −0.8/+0.6 V vs SCE interval (the scan direction was first towards negative potential values and then reversed to the anodic direction). The effect of the pH on the energetics of these trap states was determined in a similar fashion, by adjusting the pH of the borate buffer with 2 M HCl. The ionic strength of all the electrolytic solutions was maintained constant by adding 2 M NaCl in the required amount.

Determination of electrochemically active surface area (ECSA) by double layer capacitance measurements, was carried out with an Autolab PGSTAT 302 N potentiostat in a three‐electrode configuration using either GC or bare ATO as working electrodes, a Pt wire as counter electrode and a SCE as the reference, in an electrochemically inert 0.5 M borate buffer pH 9 solution. CV scans were recorded at scan rates of 10–300 mV s^−1^, in a potential range at which no faradaic processes occur. From the resulting traces, the capacitive current was evaluated as (j_a_–j_c_)/2, with j_a_ and j_c_ the anodic and cathodic current densities, respectively. When the resulting values were plotted vs. the scan rate, the slope affords the electrode double layer capacitance (C_dl(GC)_ and C_dl(ATO)_). Since the GC is assumed to be a flat electrode having geometrical area roughly corresponding to its ECSA, the ECSA of ATO can be computed according to: ECSA(ATO)=C_dl(ATO)_/C^sp^_dl(GC)_, where C^sp^_dl(GC)_ is the double layer specific capacitance of the glassy carbon disk electrode.

Current density/voltage (JV) curves were recorded with a PGSTAT 302 N potentiostat coupled with an ABET sun simulator equipped with an AM1.5G filter. The spectral irradiance was adjusted to 100 mW cm^−2^ (1 SUN) by means of a Newport 1918‐C power meter. The measurements were collected by employing ATO or ATO‐**PDI** electrodes as working electrode, SCE as reference and Pt wire as counter electrode, in the presence of a 400 nm cut‐off filter. The electrodes were illuminated from the front side.

Electrochemical impedance spectroscopy (EIS) data were recorded in the dark and under 1 SUN illumination, using a FRA2.v10 frequency response analyzer controlled by Nova 1.10. The ATO‐**PDI**‐based photoanode was polarized at the E_1/2_ of the TEMPO(+)/TEMPO couple. A 10 mV amplitude sinusoidal perturbation, whose frequency ranged between 100000 and 0.01 Hz, was applied. The equivalent circuits employed for the data analysis (inset Figure S8) included a constant phase element (CPE), that is, a nonideal capacitance accounting for the inhomogeneity of the mesoporous semiconductor. In particular, the CPE is defined by the admittance (Q) and a parameter (α), which are related to the CPE impedance (Z) as:

(2) $$Z=\frac{1}{Q\cdot {\left(j\omega \right)}^{\alpha }}$$

where j is the imaginary unit and ω is the angular frequency. Q and α can be derived by the fitting of the EIS data with the ZView software. The effective capacitance (C) can be computed according to:^[32]^

(3) $$C=Q^{\frac{1}{\alpha }}{R_{u}}^{\frac{1-\alpha }{\alpha }}$$

where R_u_ is the electrolyte uncompensated resistance between the working and the reference electrodes.

Bulk electrolysis aimed at accumulating FDCA were performed in a Nylon custom made two‐compartment cell, by separating the two sides with a Nafion‐117 membrane. The experiments were recorded with the same equipment described above, in the presence of a 400 nm cut‐off filter.

Quantification of target compounds was performed by means of high‐performance liquid chromatography with diode array detection (HPLC‐DAD). A calibration curve was generated from standard solutions of HMF, FFCA, and FDCA, by optimizing a previously described procedure.^[25]^ Briefly, prior to injection, electrolyzed solutions were filtered through 0.22 μm nylon filters and diluted 1 : 100 with Milli‐Q water. Samples were then injected into an Agilent 1100 HPLC (Agilent, Santa Clara, CA) system equipped with a binary pump, autosampler, column thermostat, and DAD detector. Chromatographic separation was achieved on a Phenomenex Luna Phenyl‐Hexyl column (100×4.6 mm, 3 μm, 100 Å) under isocratic conditions using a mobile phase composed of 3 : 97 % (v/v) methanol/0.1 M sodium formate buffer (pH 2.5, adjusted with formic acid). The injection volume was 10 μL, the column temperature was 25 °C, and detection wavelengths were set at 265 nm for FDCA and 284 nm for FFCA and HMF. Approximate retention times for HMF, FDCA, and FFCA were 6.3, 2.5, and 3.5 min, respectively.

## 2 Results and Discussions

### 2.1 General Characterization of ATO‐PDI based Films

**Figure 1:**
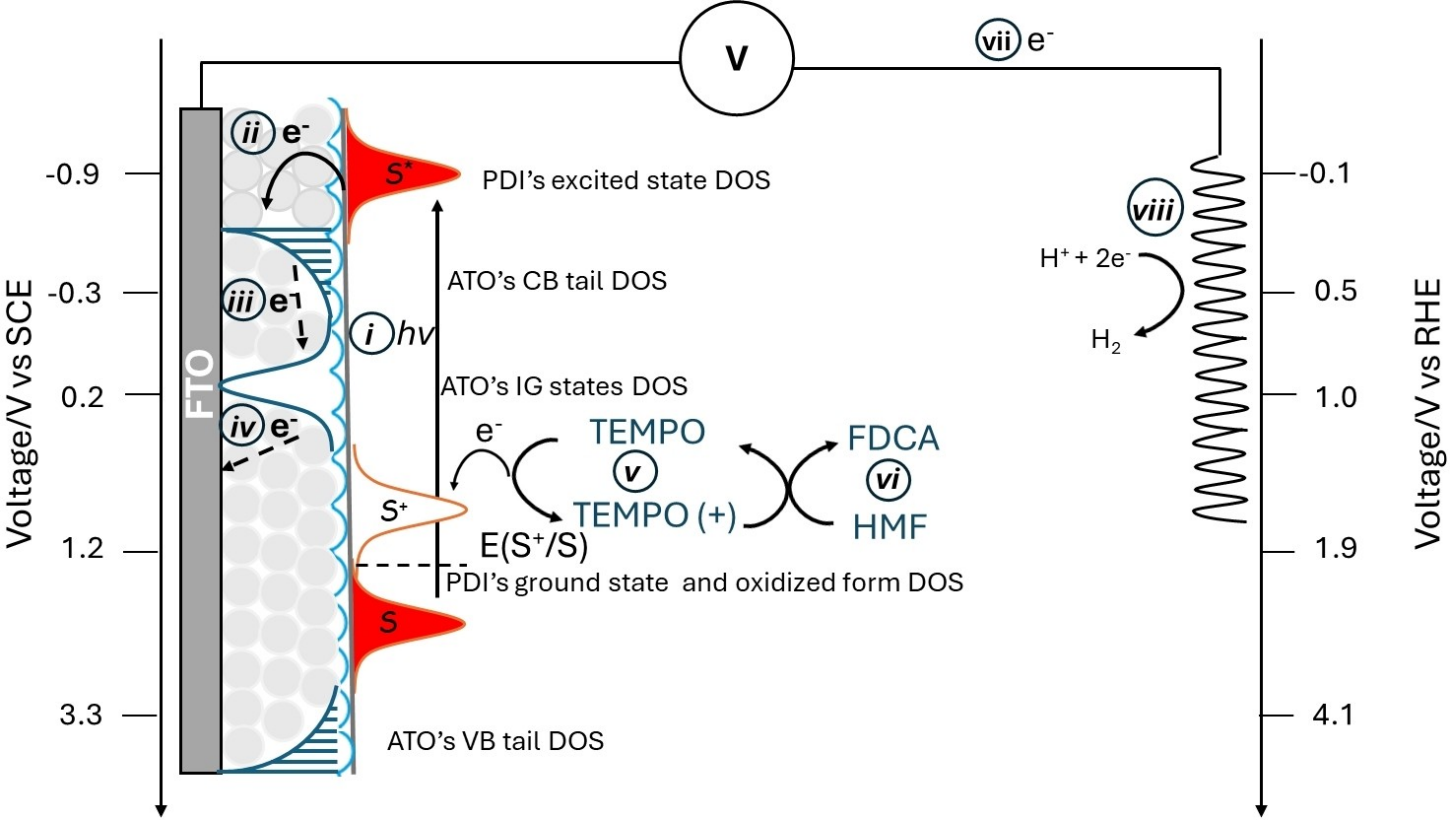
Schematic representation of the main processes occurring at the ATO‐**PDI**/electrolyte interface: i) **PDI** light absorption, ii) charge injection, iii) electron thermalization into ATO′s IG states, iv) electron collection at the ohmic collector (FTO), v) sensitizer regeneration/hole transfer to the redox shuttle (TEMPO), vi) HMF oxidation, vii) electron migration to the counter electrode and viii) proton reduction.

Tin oxide (SnO_2_) or antimony‐doped tin oxide (SnO_2_:Sb, ATO) semiconductors have been used as sensitization platform in conjunction with chromophores characterized by a relatively low excited state oxidation potential (E_ox_^*^), with the aim of ensuring a greater driving force for charge injection (ΔG_inj_) (Figure 1).[^33^, ^34^, ^35^] Among these, compared to tin oxide, ATO displays some potentially interesting features coming from the presence of intra band gap (IG) states originated from antimony doping, which result in higher conductivity and may provide better electron collection once they are emptied by applied potentials higher than 0.2 V vs SCE. These states are generated as consequence of the substitution of Sb^5+^ ions into the cassiterite structure of the SnO_2_ lattice,^[36]^ but the coexistence of Sb^3+^ and Sb^5+^ ions is often reported for ATO substrates.[^20^, ^36^, ^37^, ^38^, ^39^, ^40^, ^41^] The formation and occupancy of IG states has been detected and studied also by means of spectroelectrochemical techniques.^[20]^

Prior to ATO′s deposition, the FTO glass was treated with TiCl_4_ followed by an annealing step. This thermal process generates a thin and compact TiO_2_ blocking layer capable of passivating the back ohmic contact of the exposed FTO, with the aim of reducing charge recombination in the final DSPEC system.[^42^, ^43^] The thickness of the ATO semiconductor was evaluated by profilometry analysis, which shows a 2.39±0.21 μm thick film arising from the subsequent spin coating of the *nano*ATO suspension (Figure S1a). The top view analysis carried out by means of Scanning Electron Microscopy (SEM) on the resulting ATO electrodes confirms the nanocrystalline nature of the mesoporous semiconductor (Figure S2), consisting in a compact and polydisperse network of nanoparticles ranging between 15–30 nm (Figure S2a and b). The latter allow for large electrochemically active area resulting from permeation of the semiconductor network by the liquid electrolyte. For the sake of comparison, SEM images of undoped SnO_2_ films revealed a thickness of 4.45±0.11 μm (Figure S1b) and a morphology consisting of compact nanoparticles ranging between 10–15 nm, similar to that found in ATO (Figure S2c and d).

**Figure 2:**
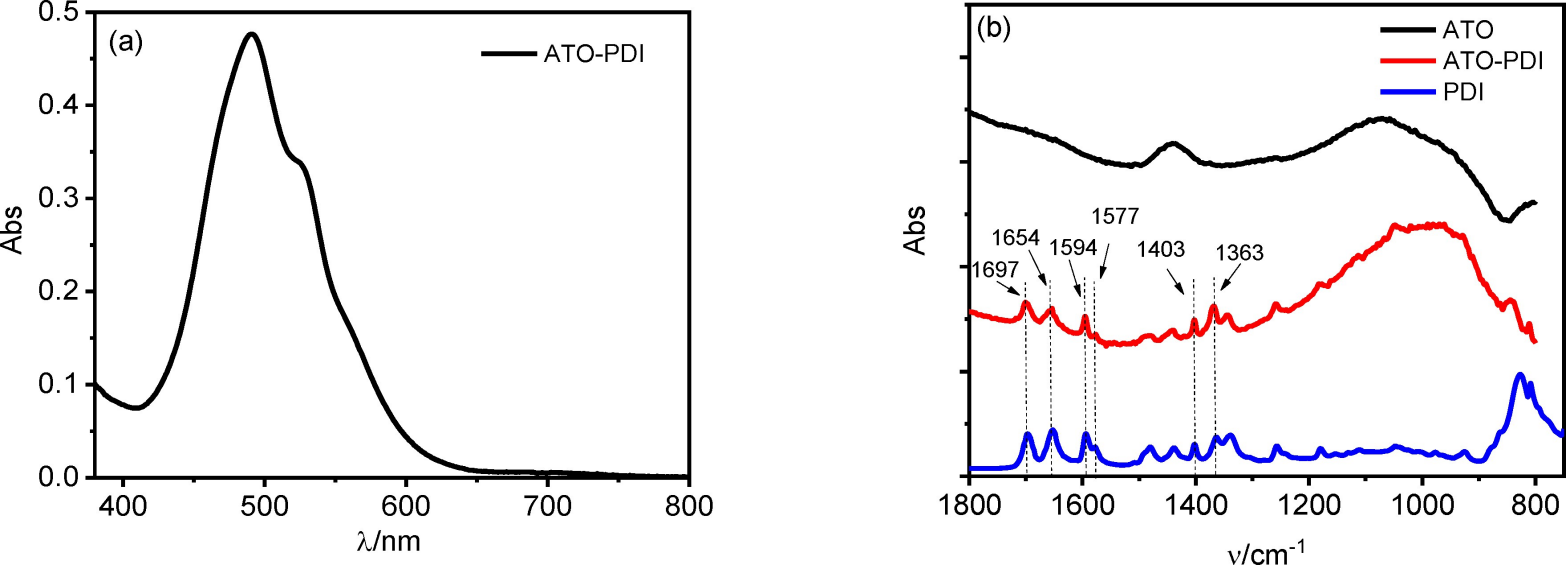
(a) Absorption spectrum for **PDI** absorbed on ATO‐based electrode. The spectrum was collected against a bare FTO. (b) FTIR‐ATR spectrum of the **PDI** (blue line) and FTIR spectra of ATO‐**PDI** (red line) and bare ATO (black line).

The absorption spectrum of the **PDI**‐sensitized ATO (Figure 2a) exhibits the expected 0–1 vibronic band peaking at λ=490 nm and a shoulder corresponding to the 0–0 transition centred at 525 nm. The latter peak is reported to be higher in intensity in the UV‐Vis spectrum of dissolved **PDI**, but an intensity reversal is reported upon its uptake onto semiconductor surfaces, due to the formation of π‐stacked hydrophobic aggregates.[^19^, ^44^, ^45^] The FTIR spectrum of the dyed photoanode (Figure 2b, red line) clearly exhibits the stretching bands of the perylenic sensitizer (Figure 2b, blue line), further confirming the capability of the dye to effectively interact with ATO′s surface, regardless the lack of traditional anchoring moieties.^[46]^ The vibrational bands at 1697 cm^−1^ and 1654 cm^−1^ can be attributed to the asymmetric and symmetric imide C=O stretching modes, while the 1403 cm^−1^ and 1363 cm^−1^ bands refer to the imide C−N stretching. The features of the perylene condensed rings can be found in the 1590–1580 cm^−1^ range while an intense band at 800 cm^−1^ could be originated by out of plane C−H bending.^[26]^ Together, the morphologic and spectroscopic characterizations allow for the synthesis of ATO‐**PDI** electrodes with significant light harvesting efficiency (ca. 67 % at λ_MAX_), instrumental for the operation of the resulting DSPEC system.

### 2.2 Electrochemical Properties of ATO and ATO‐PDI Electrodes Towards TEMPO(+)/TEMPO

**Figure 3:**
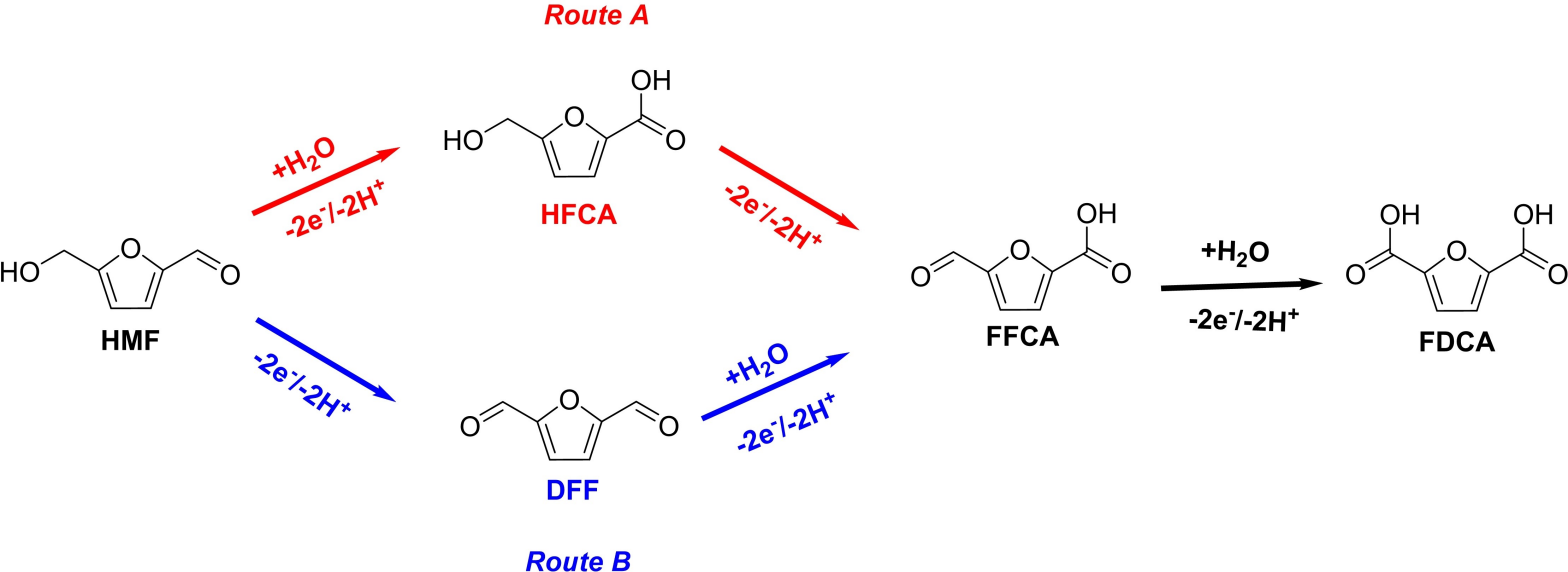
Reaction routes reported for HMF oxidation.^[21]^

The large overpotential (>1 V vs SCE) required to perform HMF oxidation (HMF+6 OH^−^→FDCA+4 H_2_O+6 e^−^) together with the need of high working pH (>13),^[47]^ can be overcome by employing TEMPO radical as a redox shuttle under relatively mild conditions, such as pH 9 and a potential >0.5 V vs SCE.[^21^, ^48^] As reported in Figure 3, HMF conversion involves the formation of partially oxidized intermediates, each one involving a bi‐electronic oxidation. The first step can proceed through two different pathways: *Route A* leads to the formation of the 5‐hydroxymethyl‐2‐furan‐carboxylic acid species (HFCA), while *Route B* to 2,5‐diformylfuran (DFF). The latter is commonly reported as the preferential route for the TEMPO mediated electrocatalytic process.[^21^, ^48^]

**Figure 4:**
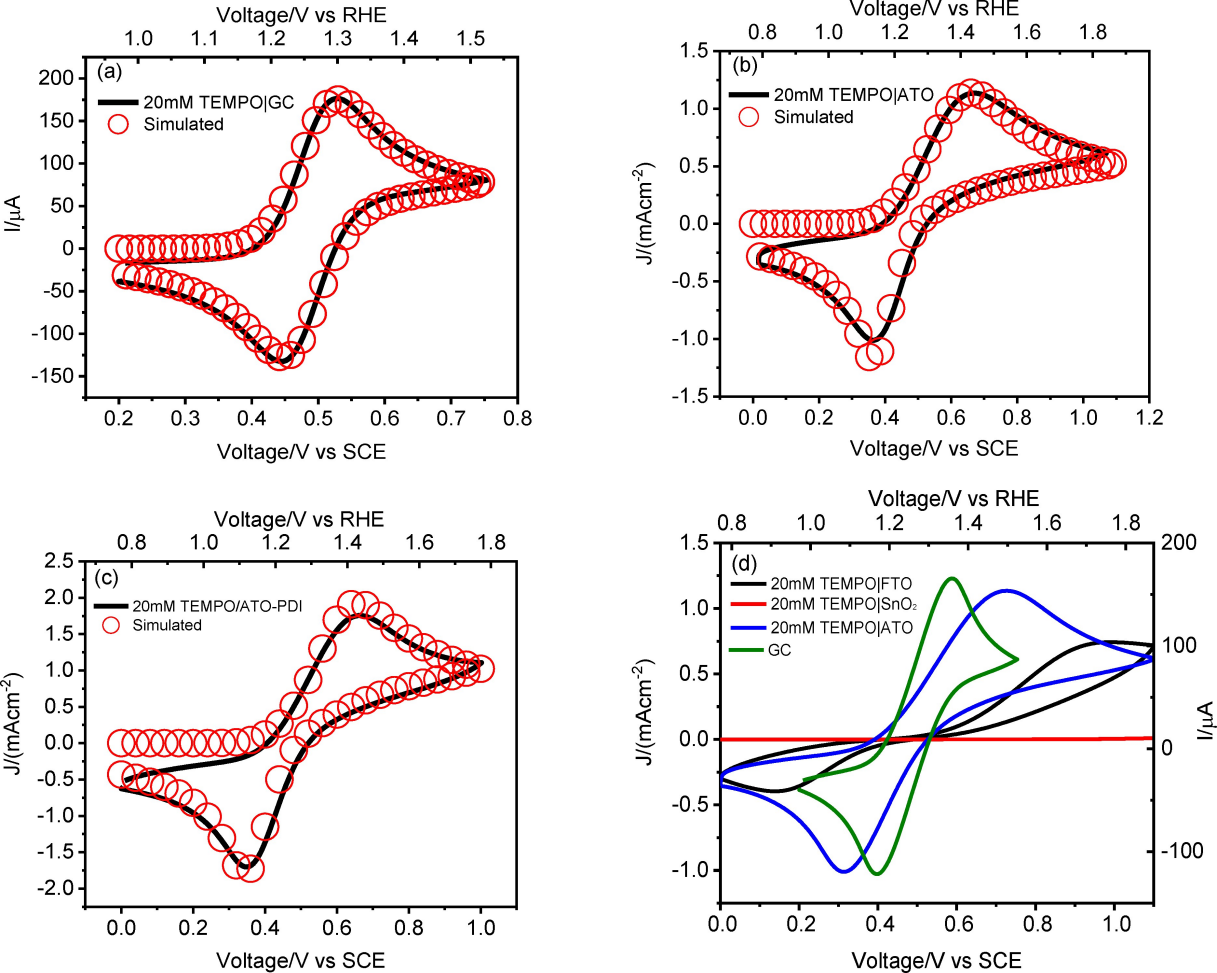
Comparison between the experimental (black line) and simulated (red circles) cyclic voltammetry for (a) GC, (b) ATO and (c) ATO‐**PDI** electrodes in a 20 mM TEMPO/0.5 M borate buffer pH 9.^1^The simulation of the baseline of the voltammetric wave in the 0.0–0.2 V vs SCE range was not in perfect agreement with the experimental curve, due to the presence of a tail of redox states in the semiconductor close to the conduction band edge of ATO. This process was not considered in the simulation, being irrelevant to the TEMPO redox chemistry. (d) Electrochemical response of a 20 mM TEMPO/0.5 M borate buffer pH 9 on FTO, SnO_2_, ATO or GC as comparison (black, red, blue and green traces respectively). The left y‐axis refers to metal oxide‐based electrodes, while the right one to GC (current values not normalized for the geometrical area).

In view of performing such reaction, we firstly evaluated the electrochemical oxidation of TEMPO by means of cyclic voltammetry in borate buffer at pH 9. The ohmic‐drop (iR) compensated voltammetric response of a 20 mM TEMPO/borate buffer pH 9 solution on a glassy carbon electrode (GC) exhibited a clear diffusion‐limited anodic wave associated with the one‐electron oxidation of TEMPO to TEMPO(+), followed by the symmetric backward feature indicating the regeneration of the reduced species, affording an E_1/2_ of 0.49 V vs SCE (Figure 4a). Overall, typical features of chemically reversible but a kinetically quasi reversible process (ΔE_peak_≈80 mV) are evident, for which ΔE_peak_ is significantly larger than the 60 mV expected for a rigorously thermodynamically reversible one electron redox reaction at T≈298 K.

In quasi‐reversible reactions, the boundary conditions to solve the diffusion equation for an electroactive species at a planar electrode are given by the Butler‐Volmer equation rather than by the Nernst equation.^[49]^ Matsuda and Ayabe^[50]^ have shown that the values of peak current and potential depend on the transfer coefficient α and on the quantity Λ, defined as:

(4) $$\Lambda =\;\frac{k^{0}}{D_{O}^{1-\alpha }D_{R}^{\alpha }fv^{1/2}}$$

where *k* ^*0*^ is the charge transfer rate constant, *D_O_* and *D_R_* are the diffusion coefficient of the oxidized and reduced form of a given electroactive species, *f=1/RT* and *ν* is the scan rate. When Λ≈10, the behavior of a quasi‐reversible system approaches that of a reversible one.

The voltammetric peak current (*i*_*peak*)_ is given by:
(5)

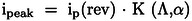




where *i_p_(rev)* is the ideally reversible current and *K*≤1 is a function of Λ and α.

In this work, we have used the electrochemical simulation software KISSA‐1D from Bioanalytical Systems to simulate the experimental voltammetric curves and obtain kinetic and mechanistic information from our electrodes. The starting point is the simple one electron electrochemical step (E) involving the oxidation of TEMPO according to (**6**), which we assumed to occur at a glassy carbon (GC) electrode:
(6)

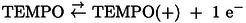




This process resulted in a satisfactory simulation of the voltammetric response of TEMPO at GC as shown by the good overlap between the experimental and computed data points in Figure 4a. The diffusion coefficient for both the reduced and oxidized forms (D_TEMPO_ and D_TEMPO(+)_) was on the order of 4.9×10^−6^ cm^2^ s^−1^, in agreement with our previous findings.^[25]^ The charge transfer coefficient (α) for the diffusion‐controlled reaction was estimated to be 0.55, further supporting the symmetrical nature of the electrochemical process, indicating that both the forward and backward steps require almost the same activation energy. It is worth nothing that the *k* ^*0*^ was estimated to be on the order of 5.8×10^−3^ cm s^−1^, in good agreement with literature data. The relevant parameters extrapolated using the electrochemical software simulation are summarized in Table 1.^[25]^

**Table 1:** Kinetic and mechanistic parameters extrapolated using the electrochemical simulation software KISSA‐1D.

Substrate	k°/(cm s^−1^)	α	k°_(ads)_/(cm s^−1^)	α_(ads)_	β_TEMPO(ads)_/M^−1^	β_TEMPO(+)(ads)_/M^−1^
GC	5.8×10^−3^	0.55	/	/	/	/
ATO	2.3×10^−4^	0.69	1.0×10^−4^	0.50	10	20
ATO‐**PDI**	2.1×10^−4^	0.63	1.0×10^−4^	0.50	2	15

The electrochemical response of TEMPO redox mediator was also evaluated using mesoporous SnO_2_ and ATO (i. e. the actual photoanodic scaffolds in the final DSPEC) as the working electrodes, as well as on FTO and GC as references (Figure 4d).

The voltammetric response of TEMPO at bare FTO revealed a slow electrochemical process, resulting in an almost irreversible TEMPO oxidation with a ΔE >1 V (black trace in Figure 4d), and an anodic wave which was nearly twice as large as the cathodic wave suggesting an asymmetric barrier for the electron transfer. This poor electrocatalytic response was supported by the software simulation, with a computed *k* ^*0*^≈10^−6^ cm s^−1^. When TEMPO oxidation was performed on SnO_2_, the electrochemical response was negligible (red trace in Figure 4d) and the electrode appeared to be almost ideally polarizable in the potential region of interest. By contrast, for the Antimony‐doped analogue, a well‐defined and quasi‐reversible process was registered, characterized by a ΔE of ≈400 mV and a current of 1.13 mA cm^−2^ (blue trace in Figure 4d). Despite the lower reversibility of the TEMPO oxidation at ATO′s surface, testified by the significantly larger peak separation compared to GC, the faradaic current in the former is larger by a factor of ca. 10 compared to the latter. This is due to the much larger electrochemically active surface area (ECSA) of the mesoporous electrode compared to GC (≈50×factor, see the CV curves registered in borate buffer pH 9, Figure S3). Thus, the possibility of using a large active area catalytic electrode which can be readily obtained through wet deposition procedures, makes the use of ATO extremely appealing for TEMPO mediated (photo)electrocatalytic processes.

**Figure 5:**
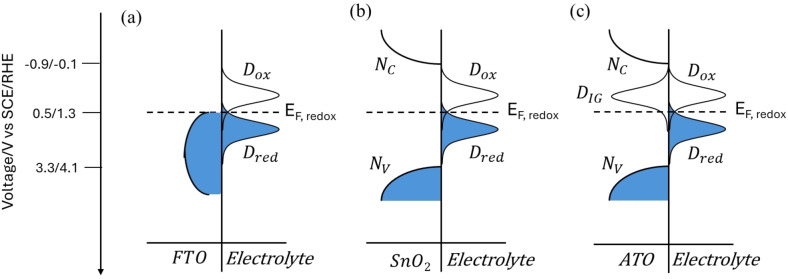
Schematic distribution of energy states (DOS) of the redox couple (TEMPO(+)/TEMPO) and (a) FTO, (b) SnO_2_, or (c) ATO under equilibrium conditions. In the case (c), a matching between ATO′s IG DOS and the E_F_ of the redox shuttle is the key for observing the electrochemical response at this semiconductor electrode.

The distinct behavior of the Sn(IV)‐based materials of Figure 4d can be rationalized in terms of the Gerischer′s model, which assumes that electron transfer can only occur between isoergonic states,^[51]^ one of which is vacant, and the other is occupied. In this framework, the anodic current *i_a_* (i. e. the current arising from TEMPO oxidation) is given by:

(7) $$i_{a}=FN\int_{-\infty }^{\infty }k\left(E\right)N_{unocc.}\left(E\right)D_{red}\left(E\right)dE$$

where *N*_*unocc*._ *(E)* and *D_red_ (E)* are the density of empty states in the electrode and of occupied energy states in the redox couple respectively, while *N* is the density of molecules that are able to reach the proximity of the electrode at a useful distance to enable charge transfer. The latter depends on *k(E)*, i. e. a coupling parameter or transient coefficient, depending on the distance between the reacting molecules at the electrode surface. The distributions of energy states in the electrolyte (*D*) are represented in Figure 5 according to a Gaussian distribution depending on the reorganization energy.^[52]^

If we consider SnO_2_ (Figure 5b), we observe that no significant density of states exists in this material in correspondence of the Fermi level E_F_ of the redox couple (approximately the E_1/2_), making the value of the integral in Equation (**7**) very small. Furthermore, the faradaic process originating by the underlying exposed FTO serving as the ohmic contact for SnO_2_ is also suppressed by the passivating TiO_x_ underlayer, thus explaining the electrochemical inertness of FTO/TiO_x_/SnO_2_ electrodes towards TEMPO oxidation. On the other hand, the energy overlap between acceptor states in the semiconductor and redox states of the electrolyte is realized with ATO electrodes (Figure 5c), since the distribution of sub‐bandgap (IG) states has been shown to extend in a broad potential region in the 0.1–0.4 V vs SCE interval; thus, a sizeable faradaic process arises. The electrochemical activity of these IG states was clearly observed by means of cyclic voltammetry in borate buffer by scanning the potential in the −0.8/+0.6 V vs SCE interval (the first scan direction was towards negative potential values, then reversed to the anodic direction). When the cathodic voltage was progressively pushed towards increasing negative values (Figure S4a), the anodic peak around 0.2 V vs SCE became correspondingly more intense and cathodically shifted at the same time. These observations are consistent with those we recently reported in acidic electrolytes (HBr, HClO_4_)^[20]^ and were interpreted, based also on spectroelectrochemical evidences, as the result of the re‐oxidation of Sb^3+^ (generated in the forward negative scan) to Sb^5+^. Importantly, while Sb^5+^ ions form a shallow donor level close to the conduction band of ATO acting as electron donors, Sb^3+^ has been reported to behave as an electron acceptor.^[53]^ Here, we have also investigated the effect of the pH on the energetics of these deep trap states, finding a convincing Nernstian‐type dependance according to: E_IG_ (pH)=E_IG_ (pH 0)–0.065×pH (Figure S4b). This evidence strongly suggests that the redox chemistry of deep IG states involves a proton coupled electron transfer step (PCET) as a probable mechanism of charge compensation. Protons may be sitting as interstitial ions, as reported in some SnO_2_ materials,^[54]^ or be exchanged through hydroxyl groups, ubiquitous at the surface of metal oxides.

Referring to TEMPO redox behavior, the simple outer sphere electrochemical process in Equation (**6**) was generally not successful in modeling its experimental voltammetric response at these ATO electrodes, affording unreasonable values of both the diffusion and the α coefficients. In particular, the voltammetric response in Figure 4b is characterized by significantly broad waves, which are suggestive of dispersion in the half wave potential[^55^, ^56^] of TEMPO. This can be due to interaction of the electroactive species with the electrode surface presenting a heterogeneous distribution of adsorption sites, which arise naturally in a high surface area mesoporous semiconductor like ATO. This led us to introduce, in parallel to the simpler outer sphere electron transfer in (**6**), a charge transfer step involving adsorbed intermediates, which appear to be physically reasonable, given the porous nature of ATO and the possibility of TEMPO to interact with the metal oxide via a variety of intermolecular interactions, including electric dipole interactions with the polar semiconductor surface, coordination of the TEMPO oxygen to hard Sn(IV) and Sb(V) centers, and hydrogen bonding. Thus, the electrochemical behavior of TEMPO at the ATO electrode was simulated by adding adsorption and desorption equilibria (**8**) and (**10**) at the surface of the mesoporous substrate, involving the TEMPO reduced and oxidized species, which were described through Langmuir isotherms of the type $\frac{\theta_{X}}{1-\theta_{X}}=\beta \left[X\right]$ , where θ_X_ is the surface coverage of the species X present in a molar concentration [X]. This allowed us to successfully simulate the experimental voltammogram, where an additional electron transfer event (**9**) involves surface adsorbed TEMPO_ads_:
(8)

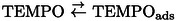



(9)

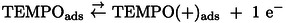



(10)

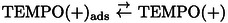




The heterogeneous outer sphere rate constant associated with (**6**) was found to be one order of magnitude lower than that extrapolated when using the GC electrode, being on the order of 2.3×10^−4^ cm s^−1^, on the same order of magnitude of process (**9**) (10^−4^ cm s^−1^), whereas the equilibrium constant of (**8**) and (**10**) are on the order of 10 M^−1^ and 20 M^−1^ respectively (Table 1). The transfer coefficient of (**6**) and (**9**) at ATO electrodes was 0.69 and 0.5 respectively, showing that, for equivalent overpotential, the oxidation of TEMPO in such conditions is faster than the reduction, mainly because of the contribution of (**6**), which is consistent with the higher intensity of the anodic wave compared to the cathodic one (Table 1). The same processes (**6**) and (**8**–**10**) modeled the response of TEMPO at ATO‐**PDI** electrodes (Figure 4c). The k^0^ associated with (**6**) was here found to be 2.1×10^−4^ cm s^−1^, slightly lower than that found in the case of the bare metal oxide, consistent with the more impeded electron transfer due to the presence of the **PDI**, while that associated to (**9**) remains on the order of 10^−4^ cm s^−1^. The transfer coefficient of (**6**) and (**9**) at ATO‐**PDI** interface was 0.63 and 0.5 respectively. The $\beta \left[X\right]$ associated with (**8**) and (**10**) decreased to 2 M^−1^ and 15 M^−1^ respectively (Table 1), consistent with a decreased affinity of TEMPO and TEMPO(+) for the sensitized electrode surface, due to both steric blockage of ATO adsorption sites by dye molecules and to electrostatic repulsions between the positively charged **PDI** and the electrogenerated TEMPO(+).

**Figure 6:**
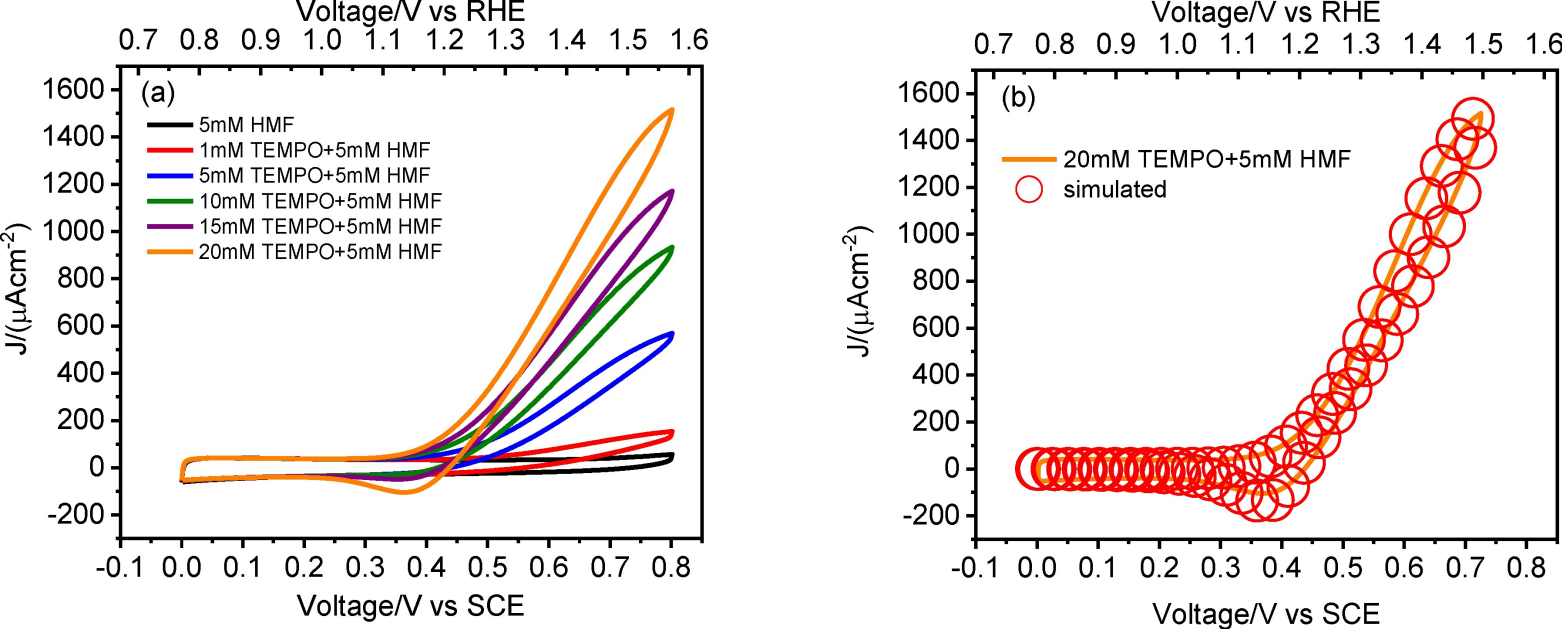
(a) Dark cyclic voltammetry for ATO‐**PDI** in contact with 5 mM HMF/0.5 M borate buffer pH 9 in the presence of increasing TEMPO concentrations from 0 mM (black line) to 20 mM (orange line). (b) Comparison between the experimental (orange line) and the simulated (circle lines) cyclic voltammetry for ATO‐**PDI** electrodes when in contact with the 20 mM TEMPO solution as described in (a) in the presence of the homogeneous chemical steps described by (**11**) and (**12**).

The TEMPO mediated HMF oxidation at ATO‐**PDI** electrodes was then evaluated by recording CVs in the presence of a constant concentration of HMF (5 mM) at increasing TEMPO concentration (from 0 mM to 20 mM, Figure 6a).

**Scheme 1:**
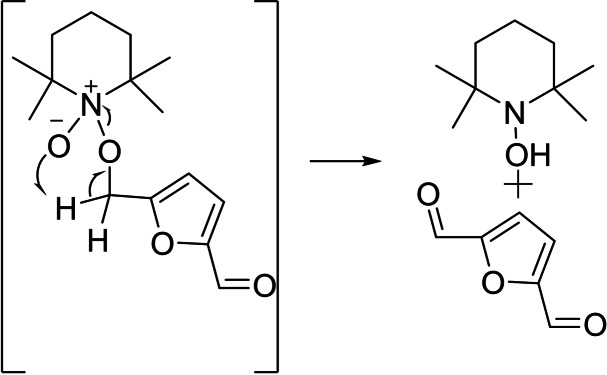
Mechanism of the primary alcohol oxidation mediated by the radical TEMPO. The formation of an oxoammonium‐alcohol adduct intermediate, followed by the intramolecular hydride transfer to provide TEMPO(H) and the formation of the new carbonyl species ( namely DFF), are highlighted.

The 5 mM concentration of HMF was chosen in order to achieve a quantitative conversion of HMF to FDCA (according to the previously described 6e^−^ oxidation mechanism) during constant potential (photo)electrolysis experiments (vide infra). Referring to Figure 6a, when the electrolyte was deprived of the TEMPO mediator, no faradaic current was recorded (black trace), confirming the need of a larger overpotential to intercept HMF oxidation in these conditions. By increasing TEMPO concentration, the catalytic current progressively increased. No backwave was observed until TEMPO concentration was three times larger that of the HMF. This behavior is expected in the presence of a so‐called EC mechanism, where the interfacial concentration of the electrocatalytic generated TEMPO(+) (E process) is effectively depleted by the subsequent chemical (C) step (namely HMF oxidation). This clearly agrees with the fact that the reaction is controlled by the electrochemical process and, in particular, by mass transport of the electrogenerated oxoammonium cation generated at the electrodic surface. By further increasing the redox mediator amount to 20 mM, the catalytic current reached 1.5 mA cm^−2^ and a partial backwave appeared (orange curve in Figure 6a). According to the most accepted primary alcohol oxidation mechanisms mediated by the TEMPO radical,^[57]^ the chemical step under alkaline conditions was firstly modelled by taking into account a bimolecular event for the formation of an oxoammonium‐alcohol adduct intermediate (k=75 M^−1^ s^−1^) (**11**). This process is followed by the intramolecular hydride transfer to provide TEMPO(H) and the formation of the DFF intermediate ((**12**) and Scheme 1).

(11) $$\mathrm{TEMPO}\left(+\right)\;+\;\mathrm{HMF}\;\vec{\leftarrow }\;\left[\mathrm{oxoammonium}\text{-}\mathrm{HMF}\right]$$

(12) [oxoammonium-HMF]→TEMPO(H)+DFF

The k_cat_ for the latter step was found to be 20 M^−1^ s^−1^, in good agreement with literature data.[^25^, ^58^] Since the ATO‐based working electrode was sensitized with **PDI**, the experimental voltammogram was stopped at 0.8 V vs SCE to avoid potential induced desorption of the positively charged dye. Thus, it cannot be ruled out that a diffusional plateau is achieved under higher applied voltage. Indeed, as reported in Figure S5a, the simulated voltammogram extended up to 1.1 V vs SCE revealed the expected sigmoidal current response (s‐shape), in which a good matching between the forward and backward scans is highlighted. This region is defined as that of pure *kinetic conditions‐no substrate consumption* ^[59]^ because the substrate concentration at the electrode is equal to the bulk concentration, leading to the efficient TEMPO regeneration once it is electrochemical generated.

The extrapolated kinetic parameters were further validated by computing the limiting catalytic current (I_p_) (**13**) generated as a consequence of the EC steps and comparing it with the experimental value:[^59^, ^60^]

(13) $$I_{p}=nFAC_{TEMPO}\sqrt{D_{TEMPO}mC_{HMF}k_{cat}}$$

where n is the number of electrons transferred per mediator molecule/unit (n=1), F is the Faraday constant, m is the number of catalyst units required per turnover (m=2 for TEMPO/HMF), and C_TEMPO_ and C_HMF_ are the bulk concentrations of the catalyst and the substrate, respectively. In the presence of 20 mM TEMPO/5 mM HMF, the I_p_ in (**13**) resulted of 1.5 mA cm^−2^, which is essentially aligned with the experimental catalytic current in Figure 6a, confirming the validity of the mechanism employed for simulating the TEMPO mediated HMF oxidation. When the HMF concentration was increased from 5 mM to 100 mM (Figure S5b), the catalytic current rose to 5.5 mA cm^−2^, which was slightly lower than the value calculated according to (**13**) of 6.7 mA cm^−2^. This discrepancy can be rationalized in terms of the iR drop, which led to an increased overpotential required to reach the anodically shifted plateau of the curve. By scanning the potential up to 1.1 V vs SCE, only the ascending branch of the current response was observed, precluding the appearance of the plateau in the s‐shaped curve.

**Figure 7:**
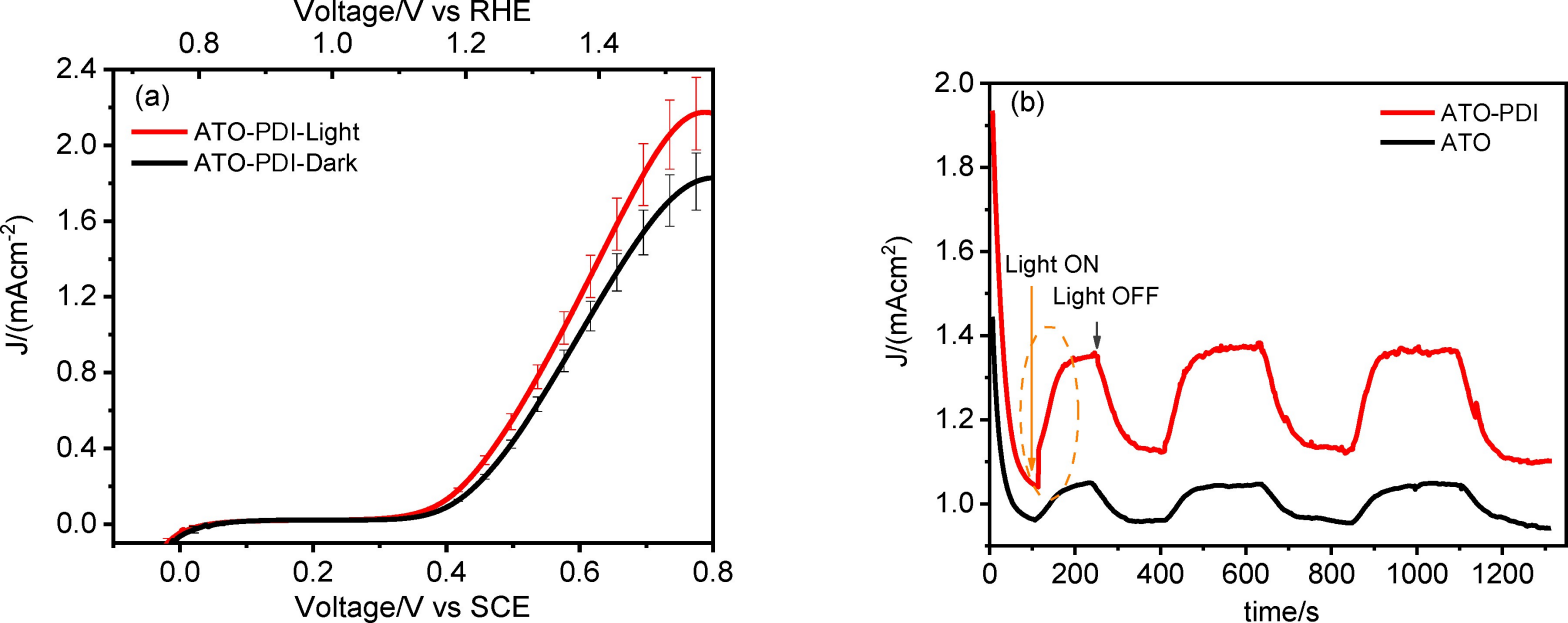
(a) JV curves for ATO‐**PDI**‐based electrodes under AM1.5G illumination (red line) and under dark conditions (black line), in contact with 20 mM TEMPO/5 mM HMF/0.5 M borate buffer pH 9 solution. (b) Chopped chronoamperometry recorded at 0.7 V vs SCE for ATO‐**PDI** (red line) and bare ATO (black line), in contact with 20 mM TEMPO/5 mM HMF/0.5 M borate buffer pH 9 solution.

Overall, this initial investigation allowed for the identification of the optimal composition of the electrolytic solution (namely 20 mM TEMPO/5 mM HMF/borate buffer pH 9, Figure 7a) to be used in the DSPEC configuration, i. e. under illumination of ATO‐**PDI** photoelectrodes.

### 2.3 Photoelectrochemical Response of ATO‐PDI Electrodes towards HMF Oxidation

Since ATO‐**PDI** electrodes are catalytically active towards TEMPO oxidation, the photoelectrochemical response of such interfaces recorded in the optimized electrolytic solution incorporates the contributions of both the dark electrochemical process promoted by ATO and the light induced electron transfer generated upon excitation of **PDI** (Figure 7a). The linear scan voltammetry in the dark shows that in such conditions (black curve) the current rises steeply for V >0.4 V vs SCE, values at which the direct TEMPO oxidation become predominant. To gain insight into the role of the **PDI** as a n‐type sensitizer, JVs were recorded under 1 SUN illumination (red curve). The current generated by the electrode under illumination is significantly higher than its dark electrochemical response, with a net gain under illumination on the order of ca. 400 μA cm^−2^, but the overall shape of the JV under AM 1.5 G parallels that obtained under dark conditions. In both cases, a plateau region seems to be almost reached at ca. 0.8 V vs SCE, which would correspond to the kinetically limited current under no substrate consumption conditions, whereas the anodic current onset under illumination is only marginally cathodically shifted with respect to the dark conditions. As discussed before, the onset of the anodic current is determined by the emptying (i. e. oxidation) of IG states, which mediate the interfacial charge transfer finally resulting in TEMPO‐catalyzed HMF oxidation. The i/t behavior (Figure 7b) recorded at 0.7 V vs SCE, shows a substantially reproducible response under irradiation/dark cycles of the duration of ca. 400 s repeated over a period of 1200 s, confirming a fair stability of the sensitized electrode under alkaline working conditions, due to the insensitivity of hydrophobic **PDI** aggregates to hydrolytic cleavage. It is interesting to observe that, compared to an otherwise identical bare ATO electrode, the **PDI** sensitized ATO systematically generated a superior current, reaching a plateau value of ca. 400 μA cm^−2^. This agrees with the comparative results from the JVs in Figure 7a. However, the kinetics leading to the attainment of such photocurrent steady state values are extremely slow (time constant in the order of 100 s) with respect to what is known from interfacial photoinduced processes. In various instances, we have investigated the relevant kinetic process in SnO_2_‐**PDI** ^[44]^ and ATO‐**PDI** sensitized electrodes, mainly applied to Br^−^ oxidation under acidic conditions, by combining various photoelectrochemical and time resolved spectroscopic methods, finding time constants from hundreds femtoseconds for injection,^[19]^ to sub millisecond for dye regeneration, to 10^−2^/10^−4^ s for charge transport across the film. Distribution of Relaxation Times (DRT) analysis also indicated that electron transport through IG states was particularly effective, resulting in a time constant of ≈10^−4^ s.^[20]^ Under no circumstances these time constants can justify the slow photocurrent dynamics herein observed. We also observe that, when ATO‐**PDI** is tested in the presence of faster light‐dark cycles (chopped illumination), the enhancement of the total current, which can be directly ascribed to TEMPO oxidation mediated by the photogenerated **PDI**(+), results much more moderate, with an intensity of ca. 20 μA cm^−2^ (Figure S6). This evidence seems to contrast with the JVs recorded under AM1.5 G continuous illumination in which a much more substantial 25 % increase in total current density was detected. The small photocurrent superimposed with the dark current observed in Figure S6 can be justified by considering that TEMPO is a fast quasi‐reversible couple, capable of undergoing fast recombination processes at the surface of ATO. Furthermore, given that ATO is catalytically active towards **PDI** oxidation, the latter is depleted from the diffusion layer of the electrode by the simultaneously occurring electrochemical oxidation reaction, ultimately resulting in a lower interfacial concentration of TEMPO capable to regenerate **PDI**, with respect to bulk values. From these observations we conclude that the enhanced current density achieved by ATO/**PDI** under illumination has to be ascribed to generation/population of an additional density of conductive IG states following charge injection by PDI*. The photopotential generated by PDI* injection into ATO is under this respect entirely analogous to a moderate forward bias, which we have shown to be effective in generating an increased density of deep sub‐bandgap states (Figure S4) which result from the interception of conduction band tail states. The long lifetime of charge carriers injected by PDI* and trapped in deep sub‐bandgap states is instrumental in achieving a permanent increase in photoanode conductivity when subjected to illumination. According to such mechanism, the photochemical generation of IG states is a slow and relatively inefficient process and manifests itself clearly only during steady state experiments (slow linear scan voltammetry under full illumination, and chronoamperometry in the 100 s time scale) partly because of rapid recombination involving photoinjected electrons and TEMPO(+), partly because of the large activation energy associated with the former. This latter condition results in a large polarization affecting the CVs in Figure S4a, where a clear Sb^5+/3+^ reductive wave is not observed (the reduction current overlaps the exponential current arising from ATO conduction band population) and in a wave separation of at least 0.6–0.7 V, that we estimate from the fact that only when the bias progresses beyond −0.4 V vs SCE a significant enhancement of the backwave centered around +0.2 V vs SCE was observed. In a similar fashion, we can explain the opposite effect, i. e. the slow drop in conductivity when dark conditions are restored in Figure 7b. Furthermore, the rise of photoconductivity effects in ATO electrodes was confirmed also testing bare photoanodes under AM 1.5 G solar irradiation; however, in such case the steady state photocurrent enhancement was smaller (≈80 μA cm^−2^, see Figures 7b and S7), since, in the absence of **PDI**, the harvesting of the solar spectrum by ATO is inefficient, limiting the production of photoinduced carriers useful to promote IG formation.

To further provide evidence of photoconduction effects arising from photochemical doping, electrochemical impedance spectroscopy (EIS) measurements were recorded for ATO‐**PDI** electrodes in contact with both TEMPO and HMF (Figure S8). The data were fitted with the equivalent circuit reported in Figure S8, in which the interface was modelled with a Randles circuit, describing the charge transfer resistance (R2=R_CT_) and non‐ideal capacitance (CPE) of a transmissive interface. The circuit incorporated also the uncompensated electrolyte and contact resistance R1, together with a Warburg element accounting for the electrolyte diffusion through the mesoporous semiconductor (Ws). The EIS data for ATO‐**PDI** recorded in dark at the E_1/2_ of TEMPO (0.49 V vs SCE) revealed a capacitance of 0.8 mF cm^−2^ and a R_CT_ of 211 Ω. The same measurement was repeated immediately after 1000 s cronoamperometry under AM1.5G illumination, during which the stationary photoconductive state was achieved: the interfacial capacitance remained essentially unchanged with respect to the dark conditions, but the R_CT_ decreased down to 131 Ω and the phase angle (θ) decreased from −30° to −20°. This behavior is consistent with a lower electrode polarization resulting from improved electrode conductivity, which enhances the charge transfer to the electrolyte. We also note that the sum of the resistances extracted from the model was in perfect agreement with $\frac{dV}{di}$ , (Figure S8c and d) showing that we can effectively reproduce the experimental iV behavior of the system through the electrical equivalent we adopted.

**Figure 8:**
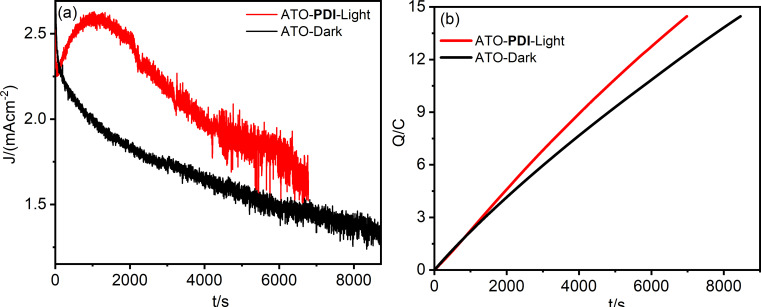
(a) Current‐time and (b) charge‐time profiles for ATO‐**PDI** (under AM1.5G illumination, red traces) and for ATO (in the dark, black traces) recorded under 0.7 V vs SCE (1.47 vs RHE) applied bias. The electrolyte was 20 mM TEMPO/5 mM HMF/0.5 M borate buffer pH 9.

To evaluate the effect of photoinduced doping on both the yield and the time required for FDCA conversion, bulk electrolysis at 0.7 V vs SCE (1.47 vs RHE) in a two‐compartment cell was performed. In the case of ATO‐**PDI** under illumination, the current‐time profile (Figure 8a, red trace) revealed a slow current enhancement, built up with a time constant on the order of 100 s, which we ascribed to the improved conductivity resulting from photochemical doping. A current maximum exceeding 2.5 mA cm^−2^ is achieved after ca. 1000 s, a value which is more than twice that observed with graphite and PEDOT modified graphite electrodes in the presence of comparable HMF concentration.^[25]^ The current is maintained at the plateau values for several hundred seconds, but after ca. 2000 s a slow drop initiates, as a consequence of the progressive consumption of HMF. By contrast, when ATO is kept under dark conditions, the current profile is solely dominated by the electrochemical process and no anodic current enhancement is observed. The lower conductivity in the dark limits the current to ca. 2 mA cm^−2^, recorded at the beginning of the experiment, followed by a progressive decay over time due to HMF consumption. Both chronoamperometry experiments were stopped when the theoretical charge of 14.47 C, required to quantitatively convert all HMF to FDCA, passed through the system (Figure 8b). It is worth noting that the mechanism of photoinduced doping of ATO enables the reaction to be completed in a 20 % shorter time scale consistent with the larger current reported in Figure 8a. Absorption spectroscopy of the anolyte solution before the electrolysis revealed a predominant band centered at 283 nm, which is ascribed to HMF, while after the prolonged chronoamperometry, the spectrum is mainly dominated by a blue‐shifted band centered at ≈261 nm, consistent with an almost quantitative conversion of the HMF substrate to FDCA (Figure S9). It is reasonable to attribute the small shoulder at 285 nm to the presence of 2,5‐formylfurancarboxylic acid (FFCA) traces. To quantitatively evaluate the Faradaic Efficiency (FE%) for FDCA production, HPLC analyses were performed. FE% were computed according to (**14**): ()

(14) $$\mathrm{FE}\;\left(\%\right)=\frac{nFDCA}{Q_{tot}/\left(F\;\cdot \;6\right)}\;\cdot 100$$

being nFDCA the number of moles of this product calculated from HPLC analysis, Q_tot_ the total charge passed during the experiment, F the Faraday constant and 6 the electrons removed in HMF oxidation to FDCA.

**Figure 9:**
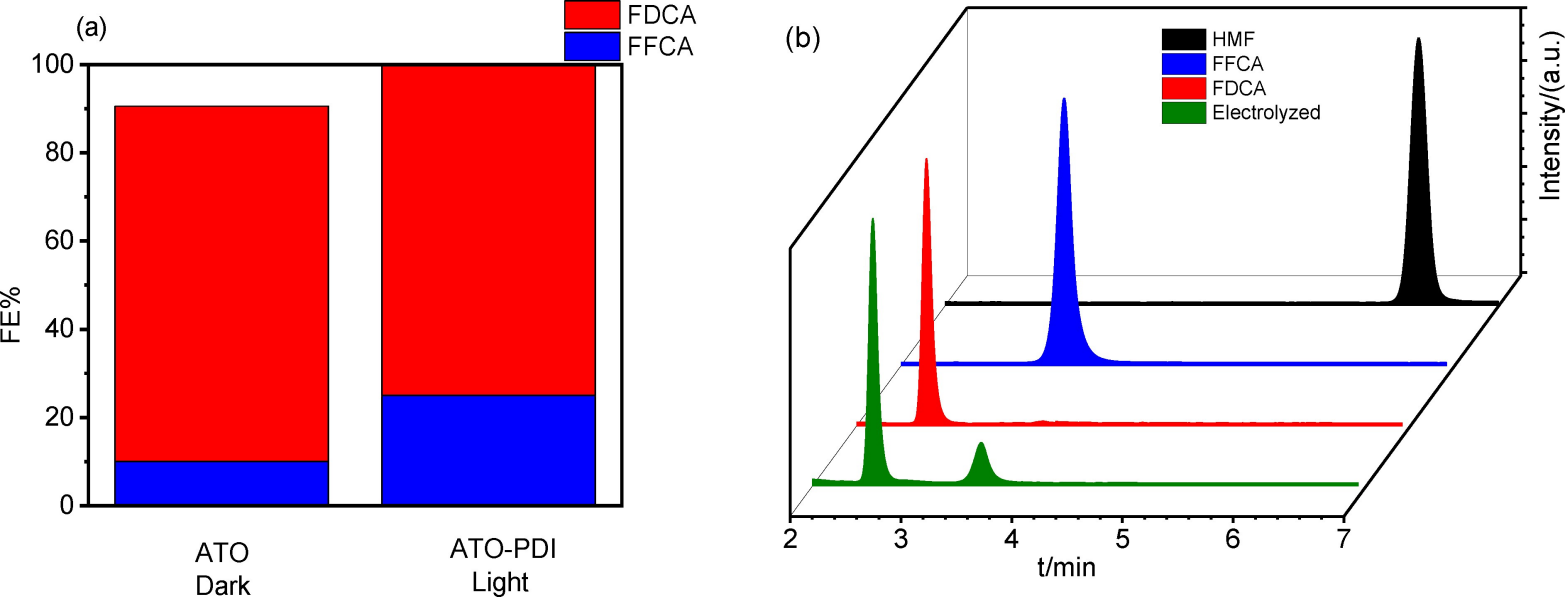
(a) Faradaic efficiency (FE%) for HMF oxidation mediated by ATO (under dark conditions) and by ATO‐**PDI** (under AM1.5G illumination). (b) Representative HPLC chromatograms for HMF (black curve), FFCA (blue curve), FDCA (red curve), and a 6 h electrolyzed solution (green curve).

Comparable FE values in the range of 75–80 % were provided by ATO under dark conditions and by ATO‐**PDI** under solar illumination (Figure 9a, Table 2). The similar FE associated to FDCA production corroborates the indication that the HMF oxidation mechanism is, in the dark and under illumination, essentially the same, and that the higher current observed under illumination has to be ascribed to enhanced photoconductivity of the semiconductor electrode. HPLC analysis confirmed the presence of some FFCA intermediate (Figure 9b). In particular, the FE for this latter process (4e^−^ involved) was of 10±7 % and 25±8 % for ATO and ATO‐**PDI**, respectively, confirming, in both conditions, the almost quantitative HMF conversion to the di‐ and mono‐carboxylated species, but also pointing at a slight superiority of the photochemically assisted process by ATO‐**PDI** yielding ca. 100 % conversion to FDCA+FFCA.

**Table 2:** Relevant parameters extracted from the bulk electrolysis.

**Substrate**	**Condition**	**Time (s)**	**FE% FFCA**	**FE% FDCA**
ATO	Dark	8463	10±7	80±2
ATO	Light	8945	/	79±2
ATO‐**PDI**	Light	6982	25±8	75±11

Control experiments have been performed to evaluate the performances of bare ATO electrodes under AM1.5 G illumination. For the reasons discussed before, the corresponding bulk electrolysis revealed a slower (time constant in the order of 1000 s) and less efficient photoconductivity effect, resulting in a steady state current which was significantly lower than in the presence of **PDI** (Figure S10). The small photochemically enhanced current did not contribute to shorten the electrolysis time substantially, thus HMF oxidation was completed in about the same time than that required under dark conditions. Coherently with the other systems, the FE% for the FDCA production was of 79±2 % (Figure S10b, Table 2); however, no FFCA was detected, likely due to possible mineralization arising from the generation strongly oxidizing photoholes produced upon direct bandgap excitation.

## 3 Conclusions

We explored the electrochemical behavior of ATO and sensitized ATO‐**PDI** electrodes in the context of the TEMPO mediated conversion of HMF to FDCA. ATO displayed good electrocatalytic properties towards TEMPO, affording a quasi‐reversible response with a heterogeneous rate constant on the order of 2×10^−4^ cm s^−1^, and providing at the same time a highly porous electrode, with a roughness factor on the order of 50 with respect to a conventional planar GC electrode. The performance of ATO under exhaustive electrolysis for the conversion of HMF to FDCA was also ca. twice superior compared to recently reported PEDOT/graphite composite electrodes. Interestingly, a significantly enhanced current was recorded over time when ATO‐**PDI** was exposed to prolonged visible light illumination, which we rationalized as the result of photoinduced doping of ATO due to the oxidative quenching of **PDI** excited states. This process translates into increased ATO film conductivity and, consequently, into current values enhanced by a factor of 20–25 % with respect to otherwise identical dark conditions. The proposed system enabled the production of FDCA (a value‐added substrate for alternative polymer synthesis) with ca. 75 % FE in <2 h reaction time, whereas the yield was quantitative (ca. 100 %) if the mono‐ and di‐ acid (FFCA+FDCA) products were considered. Work is in progress towards the fabrication of ATO electrodes incorporating conductive carbon nanostructures, with the double aim of yielding more conductive interfaces and further stabilizing the hydrophobic **PDI** aggregates, respectively resulting in higher current densities and dye loadings.

## Conflict of Interests

The authors declare no conflict of interest.

4

## Supporting information

As a service to our authors and readers, this journal provides supporting information supplied by the authors. Such materials are peer reviewed and may be re‐organized for online delivery, but are not copy‐edited or typeset. Technical support issues arising from supporting information (other than missing files) should be addressed to the authors.

Supporting Information

## Data Availability

The data that support the findings of this study are available from the corresponding author upon reasonable request.
