# Supplementary material for: 5‐Hydroxymethyl Furfural Oxidation by Perylene Diimide‐Sensitized Electrodes Boosted by Photoinduced Doping
Source: ChemSusChem. 2024 Nov 12;18(4):e202401782. doi: 10.1002/cssc.202401782 (PMC11826125; doi:10.1002/cssc.202401782)
Supplement: Supplementary file 1 — Supporting Information [file CSSC-18-e202401782-s001.pdf]

ChemSusChem

Supporting Information

5-Hydroxymethyl Furfural Oxidation by Perylene Diimide-Sensitized Electrodes Boosted by Photoinduced Doping

Edoardo Marchini,* Stefano Carli, Davide Barboni, Martina Catani, Alberto Cavazzini, Stefano Caramori, and Serena Berardi*

5-Hydroxymethyl Furfural Oxidation by Perylene Diimide-Sensitized Electrodes Boosted by Photoinduced Doping

Edoardo Marchini,[1] Stefano Carli,[2] Davide Barboni,[1] Martina Catani,[1] Alberto Cavazzini,[1,3] Stefano Caramori,[1,4] and Serena Berardi[1*]*

[1]Department of Chemical, Pharmaceutical and Agrarian Sciences, University of Ferrara, 44121 Ferrara, Italy
[2]Department of Environmental and Prevention Sciences, University of Ferrara, 44121 Ferrara, Italy
[3]Council for agricultural research and economics - CREA, 00184 Rome, Italy
[4]National Interuniversity Consortium of Materials Science and Technology (INSTM), University of Ferrara Research Unit, 44121 Ferrara, Italy

Supporting Information

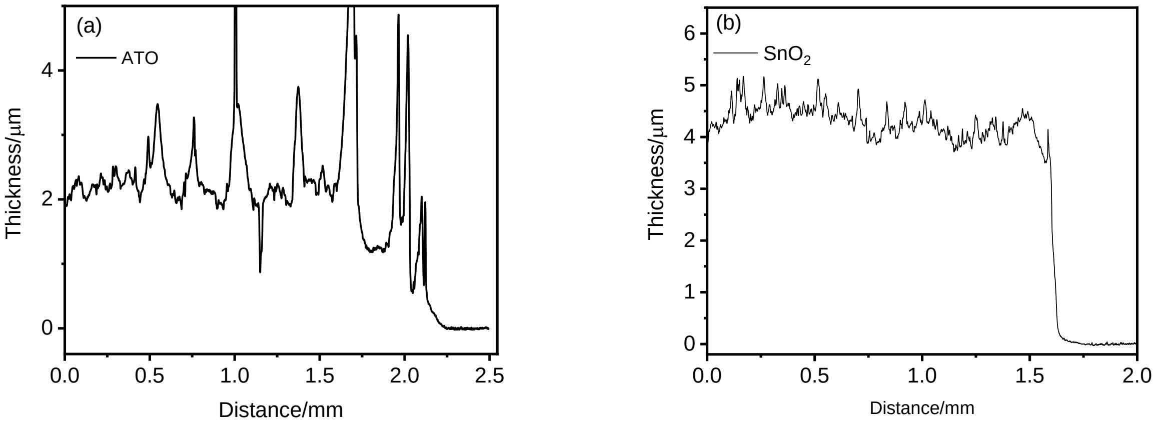

Figure S1: Representative profilometry analysis for (a) ATO- and (b) SnO_2-based electrodes. The most intense spikes in (a) are related to the presence of border effects related to the film preparation via multiple layers or to surface aggregates.

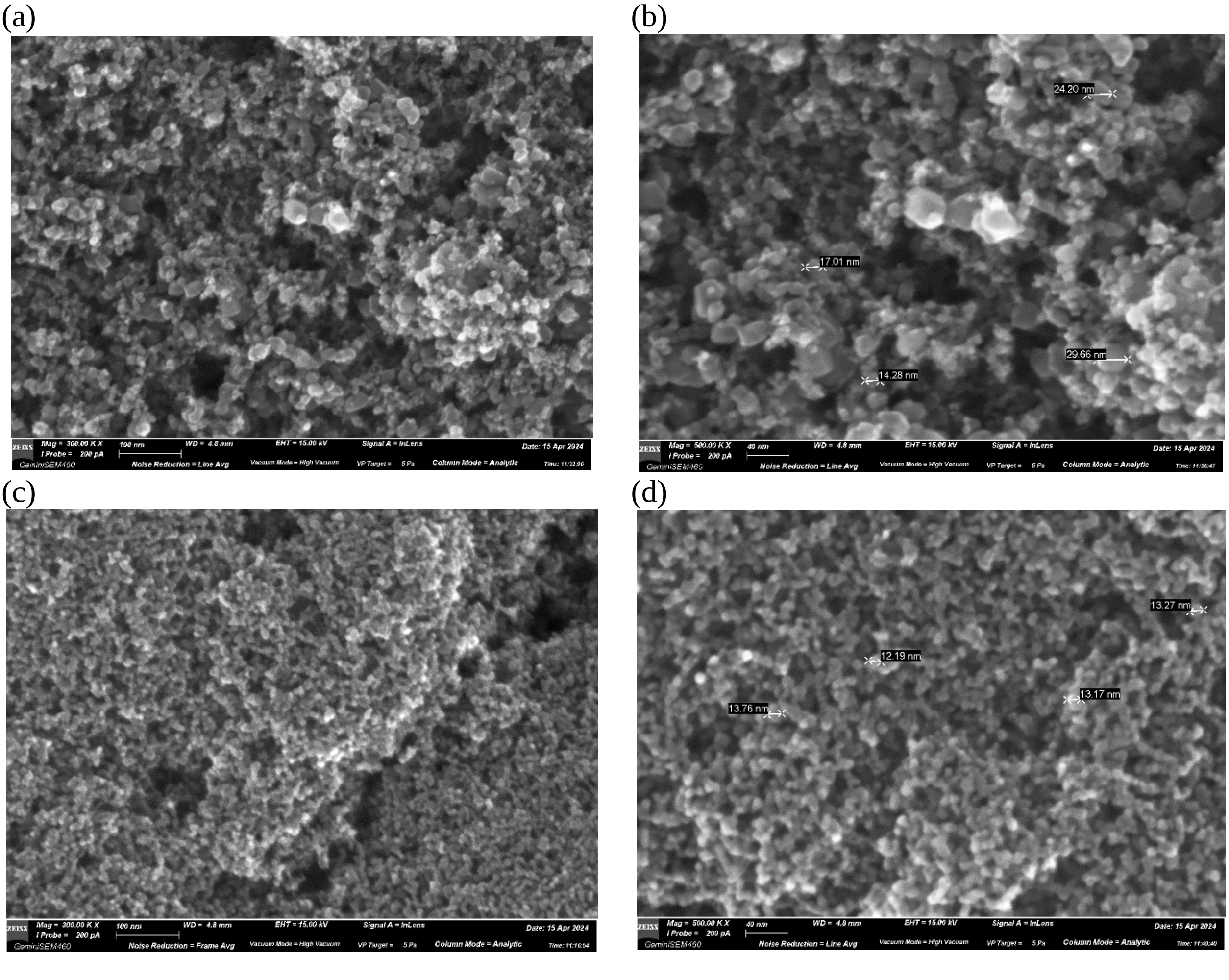

Figure S2: SEM images for (a,b) ATO and (c,d) SnO_2 electrodes. The images were collected at 300K and 500K magnification.

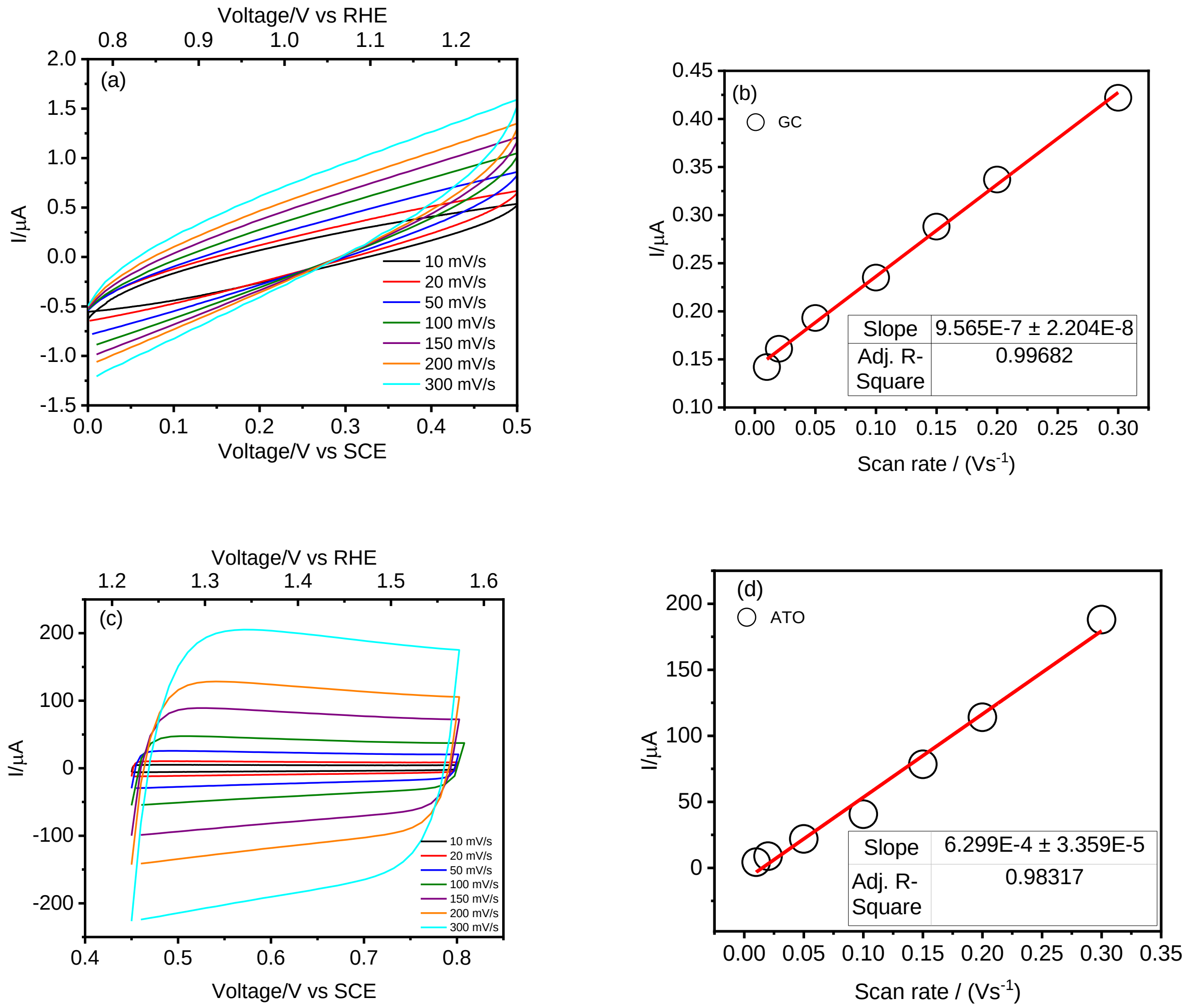

Figure S3: CV scans for the electrochemical double layer determination of (a) GC and (c) ATO in contact with a 0.5 M borate buffer solution pH 9. The corresponding linear fits for the determination of the electrode capacitance are reported respectively in (b) and (d), together with the fitted slope values in μF. By normalizing these values for the geometrical areas of the electrodes (i.e. 0.0706 and 1 cm^2, respectively for GC and ATO), $C^{sp}_{dl(GC)}$ and $C_{dl(ATO)}$ were calculated to be 13.5 μF cm^{-2} and 629 μF cm^{-2}.

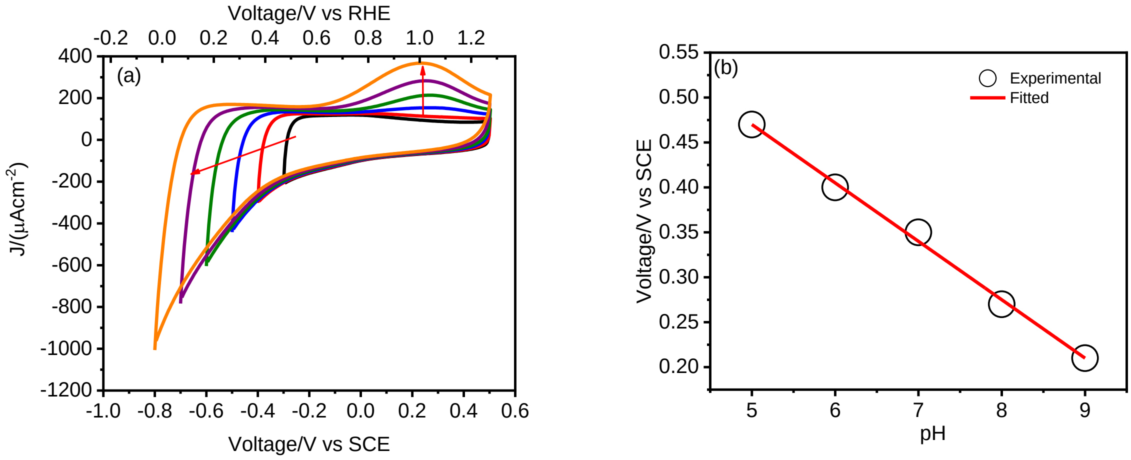

Figure S4: (a) CV scans for ATO-based electrode in contact with a 0.5 M borate buffer solution pH 9, at 50 mVs^{-1} scan rate, towards progressively more cathodic potential. (b) Potential value (E_{peak}) of IG states as a function of the pH of the borate electrolyte.

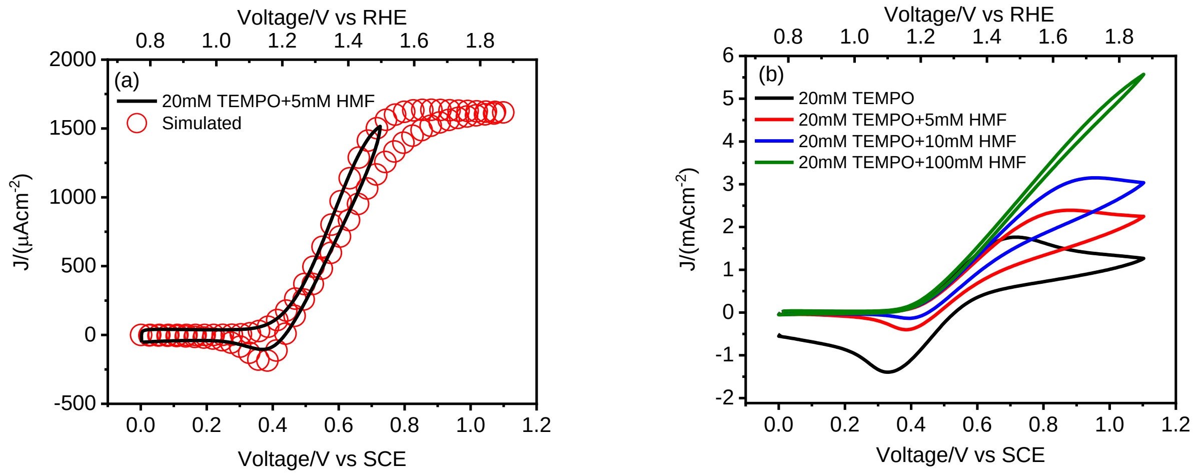

Figure S5: (a) Experimental (black line) and simulated (red circles) cyclic voltammetry of ATO-**PDI** electrodes of a 20 mM TEMPO/5 mM HMF/0.5 M borate buffer pH 9 solution. The simulated profile was extended to 1.1 V vs SCE to highlight the classical s-shape. (b) Dark cyclic voltammetry for ATO in contact with 20 mM TEMPO/0.5 M borate buffer pH 9 in the presence of increasing HMF concentrations from 0 mM (black line) to 100 mM (green line). To avoid potential induced dye desorption, the CVs were conducted in the absence of the sensitizer.

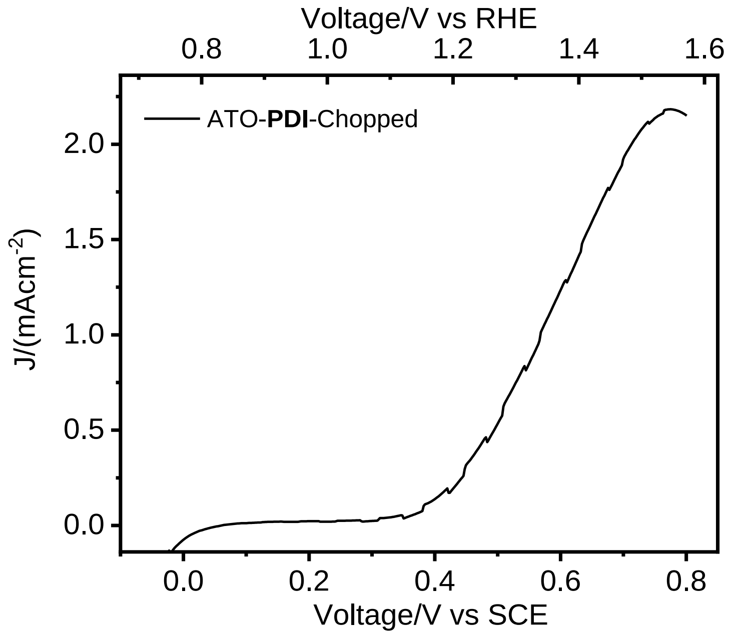

Figure S6: Chopped linear sweep voltammetry for ATO-**PDI** electrodes in contact with 20 mM TEMPO/5mM HMF/ 0.5 M borate buffer pH 9 solution.

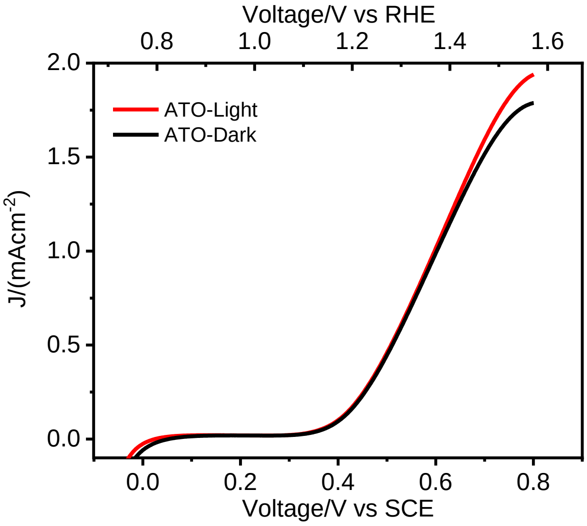

Figure S7: JV curves for bare ATO-based electrodes under AM1.5G illumination (red line) and under dark conditions (black line), in contact with 20 mM TEMPO/5mM HMF/ 0.5 M borate buffer pH 9 solution.

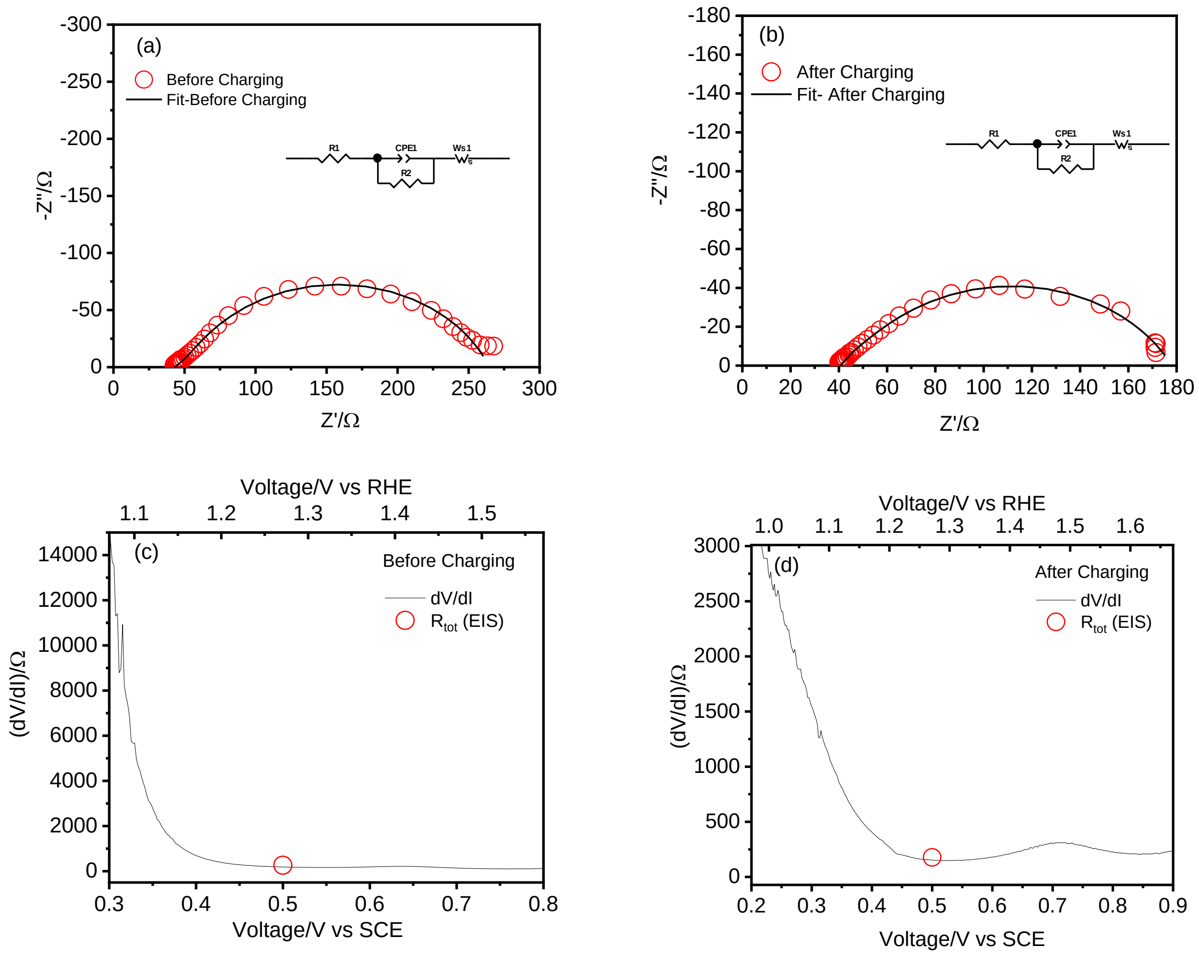

Figure S8: Nyquist plots for ATO-**PDI** in contact with a 20 mM TEMPO/5 mM HMF/0.5 M borate buffer pH 9 solution, (a) in dark before illumination and (b) under AM 1.5G illumination after 1000 s chronoamperometry at 0.7 V vs SCE under 1 SUN (which corresponds to the end of the charging process). The employed equivalent circuit is reported as insert. Reciprocal of the derivative of the JV curves (black line) ($R=\partial V/\partial I$) and total resistance extrapolated by fitting the EIS data (red dot) (c) before charging and (d) after charging.

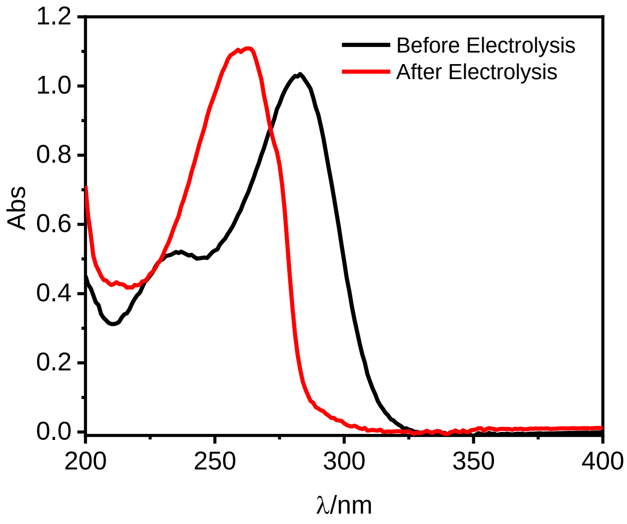

Figure S9: Absorption spectra of the anolyte before and after electrolysis.

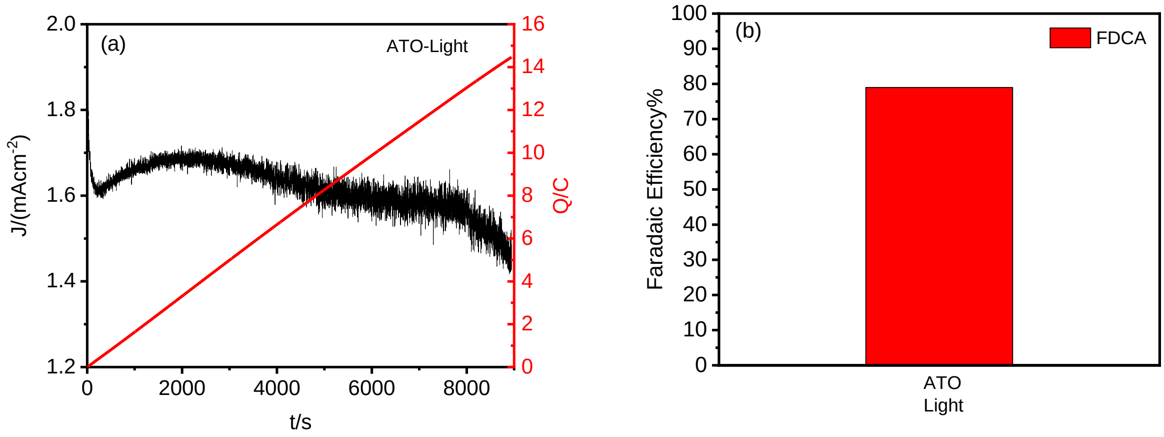

Figure S10: (a) Current-time and charge-time profile for ATO-based electrodes (under AM1.5G illumination). The electrolyte was 20 mM TEMPO/5 mM HMF/0.5 M borate buffer pH 9. (b) Average Faradaic efficiency for the experiment reported in (a).
